# A Deep Dive into the Globin Superfamily of Sharks, Skates, and Rays: Contrasting Patterns of Gene Loss and Retention Relative to Bony Vertebrates

**DOI:** 10.1093/gbe/evag058

**Published:** 2026-03-13

**Authors:** Hunter K Walt, Joseph A Hinton, Shigehiro Kuraku, Juan C Opazo, Jay F Storz, Federico G Hoffmann

**Affiliations:** Department of Biochemistry, Nutrition, and Health Promotion, Mississippi State University, Mississippi State, MS 39762, USA; Department of Biochemistry, Nutrition, and Health Promotion, Mississippi State University, Mississippi State, MS 39762, USA; Molecular Life History Laboratory, Department of Genomics and Evolutionary Biology, National Institute of Genetics, Mishima, Shizuoka, Japan; Department of Genetics, Sokendai (Graduate University for Advanced Studies), Mishima, Shizuoka, Japan; Escuela de Medicina, Facultad de Medicina, Universidad San Sebastián, Valdivia, Chile; Integrative Biology Group, Valdivia, Chile; School of Biological Sciences, University of Nebraska, Lincoln, NE 68588, USA; Department of Biochemistry, Nutrition, and Health Promotion, Mississippi State University, Mississippi State, MS 39762, USA; Institute for Genomics, Biocomputing & Biotechnology, Mississippi State University, Mississippi State, MS 39762, USA

**Keywords:** globins, cartilaginous fish, chondrichthyes, gene family evolution

## Abstract

The globin gene superfamily encodes oxygen-binding proteins that are present in all domains of life. Hemoglobin and myoglobin of jawed vertebrates are among the most well-studied proteins in the context of structure–function relationships and evolution after gene duplication. However, these studies have primarily focused on bony vertebrates, and research on the evolution of the globin gene family in cartilaginous fish has been limited by a lack of genomic resources. In this study, we leverage newly available cartilaginous fish genomes to investigate globin gene family evolution across skates, rays, sharks, and sawfish. We found that, when present, most globin genes are in a single copy, with androglobin, globin-Y, and myoglobin present in all cartilaginous fish, whereas the two globin-X paralogs of gnathostomes have been differentially retained by elasmobranchs (sharks, skates, and rays), which retained paralog 1, and the Holocephali, which retained paralog 2. Neuroglobin appears to have been lost at the common ancestor of all cartilaginous fish. The α- and β-globin gene subfamilies underwent independent expansions in different lineages of cartilaginous fish. Most cartilaginous fish globins have conserved synteny with other jawed vertebrates except myoglobin. Additionally, *Nprl3*, which directly flanks the hemoglobin clusters of other jawed and jawless vertebrates and regulates hemoglobin gene expression, is on a separate chromosome from the hemoglobin clusters of cartilaginous fish. When we examined globin gene expression patterns across cartilaginous fish tissues and developmental stages, we found that most globins are expressed as expected compared to other jawed vertebrates. However, hemoglobin paralogs are more widely expressed in embryonic tissues compared to later-stage tissues in cases where many copies exist. Our results reveal similar and contrasting patterns of globin gene evolution between cartilaginous and bony vertebrates and shed light on the early stages of globin gene evolution in gnathostomes.

Significance The evolution of the globin gene family in jawed vertebrates is of significant interest; however, most studies have been limited to bony vertebrates. With the influx of new publicly available cartilaginous fish genomes, we conducted the most comprehensive analysis of globin gene evolution in cartilaginous fish to date using phylogenetic, structural, and transcriptomic analyses. Our results shed light on similar and contrasting patterns of globin-gene evolution between cartilaginous and bony vertebrates and provide insight into the early evolution of globin in jawed vertebrates.

## Introduction

Globins are oxygen-binding proteins present in Archaea, Bacteria, and Eukarya (Vinogradov et al. 2005; Storz 2019). They share a similar structure of a heme prosthetic group sandwiched between α-helices, called the “globin fold.” The hemoglobin and myoglobin proteins of jawed vertebrates have made fundamental contributions to our understanding of structure–function relationships and the role of gene duplication in promoting functional innovation. Horse hemoglobin and whale myoglobin were the first proteins to have their crystal structures solved (Kendrew et al. 1958; Perutz et al. 1960), and sequence similarities among the human α- and β-globin chains led Ingram to propose that the subunits of tetrameric hemoglobin and myoglobin emerged via duplication and divergence of an ancestral single-copy gene (Ingram 1961). The discovery of cyclostome hemoglobins, which are monomeric when bound to oxygen and assemble into dimers or tetramers in the deoxy state (Brittain and Wells 1986), fit reasonably well with the scenario proposed by Ingram, with the dimeric hemoglobin of jawless vertebrates occupying an intermediate position between the proposed ancestral monomeric hemoglobin and the tetrameric hemoglobins of jawed vertebrates (Goodman et al. 1975; Müller et al. 2003).

Up until the end of the 20th century, myoglobin, α-hemoglobin, and β-hemoglobin of jawed vertebrates and the hemoglobins and myoglobins of jawless fish were the only known vertebrate globins. The advent of comparative genomics resulted in a new appreciation of the diversity of the globin gene superfamily in the animal kingdom (Burmester et al. 2000, 2002; Kugelstadt et al. 2004; Roesner et al. 2005; Fuchs et al. 2006; Hoffmann et al. 2010, 2012b; Opazo et al. 2015a). Phylogenetic and synteny analyses revealed that vertebrate globins diversified via a combination of tandem gene duplications and whole-genome duplications (Hoffmann et al. 2012a, 2021; Opazo et al. 2013; Storz et al. 2013). Currently, the globins of vertebrates can be separated into four groups: Androglobin (*Adgb*), Neuroglobin (*Ngb*), globin-Xs (*GbX1* and *GbX2*), and the vertebrate-specific globins. The latter group includes cytoglobin (*Cygb*), globin-E (*GbE*), globin-Y (*GbY*), the myoglobin (*Mb*), α-globins (*HbA*), and β-globins (*HbB*) of gnathostomes (jawed vertebrates) and the hemoglobins (*cHb*) and myoglobins (*cMb*) of cyclostomes (jawless vertebrates).

Many of these newly discovered globins do not have canonical oxygen-transport or oxygen-storage functions, functions that evolved independently in gnathostomes and cyclostomes, respectively (Hoffmann et al. 2010; Schwarze et al. 2014). The pro-ortholog of the *cMb* and *cHb* genes of cyclostomes is closely related to *Cygb*, whereas the *HbA* and *HbB* gene families of gnathostomes are more closely related to *Mb* and *GbE* (Hoffmann et al. 2010). The last common ancestor of vertebrates included copies of *Adgb*, *GbX1*, *GbX2*, *Cygb*, *Ngb*, *GbY*, the preduplication progenitor of the *GbE* and *Mb* genes, the progenitor of the α- and β-globin gene families of gnathostomes, and the progenitor of the *cMb* and *cHb* genes of cyclostomes (Storz et al. 2013). Lineage-specific duplications and deletions of members of this ancestral gene set account for the observed variation in the globin gene repertoires among contemporary vertebrates (Storz et al. 2013).

The early stages of globin evolution in gnathostomes have received significant interest and remain poorly understood. Morris Goodman and Motoo Kimura argued about the role of Darwinian selection on the early divergence between *Mb* and *Hb* (Goodman et al. 1975; Goodman 1981; Kimura 1981), and the branching of vertebrate-specific globins has proven hard to resolve. This is particularly true for the globin gene repertoire of cartilaginous fishes (Class Chondrichthyes), largely due to a relative dearth of genomic sequence data in comparison to resources available for bony vertebrates. The split between bony fishes and cartilaginous fishes is the deepest in the tree of extant jawed vertebrates, dating back to ∼460 million years ago (mya) (Kumar et al. 2022). Within extant cartilaginous fishes, the split between Elasmobranchii (sharks and batoids) and Holocephali (chimaeras and ratfishes) is the deepest, timed to ∼415 mya, whereas the split between sharks, superorder Selachimorpha, and batoids, which comprise rays, skates, torpedoes, and sawfish in the superorder Batoidea, is timed to ∼270 mya (Kumar et al. 2022). Until recently, research into the globin repertoire of cartilaginous fishes had been limited to the elephant fish, *Callorhinchus milii*, a representative of the subclass Holocephali that has one of the smallest globin repertoires among vertebrates (Opazo et al. 2015a). After a long period with no additional genomic resources, chromosome-level assemblies of elasmobranch genomes are now available for diverse species, which presents an excellent opportunity to expand our understanding of the evolution of the globin gene family in this group.

Here, we combine bioinformatic searches with phylogenetic and synteny analyses to characterize the globin repertoires in the genomes of sharks, rays, skates, and sawfish. We also took advantage of available transcriptome data to compare patterns of gene expression across multiple tissues and developmental stages. We report new findings regarding the differential retention of ancestral globin genes in cartilaginous fishes relative to bony vertebrates.

## Results and Discussion

### Bioinformatic Searches

**Fig. 1.**
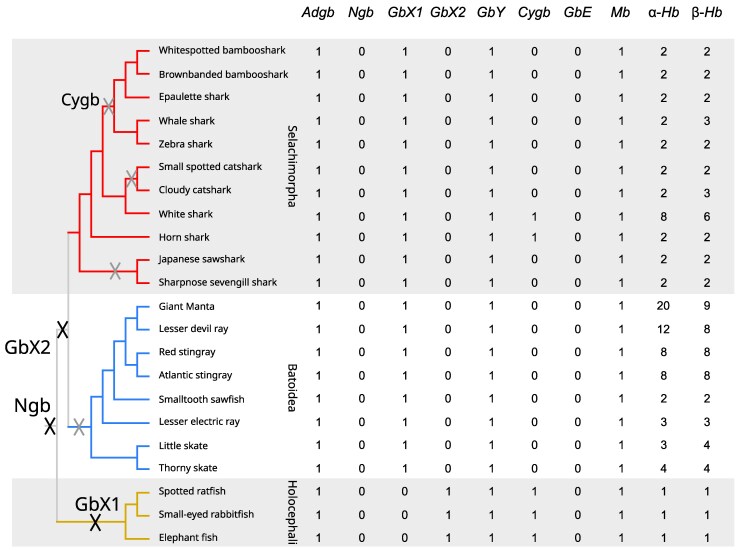
Phyletic distribution of the different globin subfamilies in the genomes of the cartilaginous fishes in our study. The counts correspond to the number of globin genes after data curation. Black Xs along the tree correspond to inferred losses of *Ngb*, *GbX1*, *GbX2*, and gray Xs refer to the repeated loss of *Cygb*. Losses were inferred by mapping genes to the last common ancestor of the species where the gene is not present.

We identified a total of 261 globin sequences from 22 species of cartilaginous fishes, 22 of which were *Adgbs* (Fig. 1, Table 1, Table S1, Supplementary material online). Our searches revealed that almost all genomes screened had single copies of *Adgb*, *GbY*, and *Mb*; that most genomes possessed multiple copies of α- and β-globin, and that *Cygb* was present only in horn shark and elephant fish (Fig. 1). Our initial searches were limited to 20 Ref-seq genomes (Table 1), where we identified 243 previously annotated globins. To this data set, we added the Adgb and Cygb amino acid sequences of the white shark, the GbY amino acid sequence of the red stingray, the GbX1 amino acid sequence of the smalltooth sawfish, and the globin repertoires of the rabbitfish and the ratfish using manual annotations. Although the Adgbs of white shark, ratfish, and rabbitfish were found in the corresponding genomes, the hits cover approximately 80% of the translated protein and are clearly incomplete. We infer that these genes are present and potentially functional, but not fully covered in the current genome releases.

**Table 1:** Cartilaginous fish genomes surveyed in the study

Database	Common Name	Species Name	Superorder	Order	Accession
RefSeq	Whitespotted bambooshark	*Chiloscyllium plagiosum*	Selachimorpha	Orectolobiformes	GCF_004010195.1
RefSeq	Brownbanded bambooshark	*Chiloscyllium punctatum*	Selachimorpha	Orectolobiformes	GCF_047496795.1
RefSeq	Epaulette shark	*Hemiscyllium ocellatum*	Selachimorpha	Orectolobiformes	GCF_020745735.1
RefSeq	Whale shark	*Rhincodon typus*	Selachimorpha	Orectolobiformes	GCF_021869965.1
RefSeq	Zebra shark	*Stegostoma tigrinum*	Selachimorpha	Orectolobiformes	GCF_030684315.1
RefSeq	Horn shark	*Heterodontus francisci*	Selachimorpha	Heterodontidae	GCF_036365525.1
RefSeq	Smaller spotted catshark	*Scyliorhinus canicula*	Selachimorpha	Carcharhiniformes	GCF_902713615.1
RefSeq	Cloudy catshark	*Scyliorhinus torazame*	Selachimorpha	Carcharhiniformes	GCF_047496885.1
RefSeq	Great white shark	*Carcharodon carcharias*	Selachimorpha	Carcharhiniformes	GCF_017639515.1
RefSeq	Japanese sawshark	*Pristiophorus japonicus*	Selachimorpha	Pristiophoriformes	GCF_044704955.1
RefSeq	Sharpnose sevengill shark	*Heptranchias perlo*	Selachimorpha	Hexanchiformes	GCF_035084215.1
RefSeq	Giant manta	*Mobula birostris*	Batomorphi	Myliobatiformes	GCF_030028105.1
RefSeq	Lesser devil ray	*Mobula hypostoma*	Batomorphi	Myliobatiformes	GCF_963921235.1
RefSeq	Red stingray	*Hemitrygon akajei*	Batomorphi	Myliobatiformes	GCA_048418815.1
RefSeq	Atlantic stingray	*Hypanus sabinus*	Batomorphi	Myliobatiformes	GCF_030144855.1
RefSeq	Smalltooth sawfish	*Pristis pectinata*	Batomorphi	Rhinopristiformes	GCF_009764475.1
RefSeq	Lesser electric ray	*Narcine bancroftii*	Batomorphi	Torpediniformes	GCF_036971445.1
RefSeq	Little skate	*Leucoraja erinaceus*	Batomorphi	Rajiformes	GCF_028641065.1
RefSeq	Thorny skate	*Amblyraja radiata*	Batomorphi	Rajiformes	GCF_010909765.2
GenBank	Spotted ratfish	*Hydrolagus colliei*	Holocephali	Holocephali	GCA_035084275.1
GenBank	Small-eyed rabbitfish	*Hydrolagus affinis*	Holocephali	Holocephali	GCA_012026655.1
RefSeq	Elephant fish	*Callorhinchus milii*	Holocephali	Holocephali	GCF_018977255.1

### Data Curation

There are two *GbX* genes in the cloudy catshark genome, NCBI Gene IDs 140406215 and 140406187, both in short contigs that do not include any additional annotated genes. These two genes are predicted to encode proteins of 152 and 136 amino acids that are identical over the positions they share. We infer that they are alleles rather than duplicate genes, and we retained the more complete sequence for downstream analyses. In the horn shark, we found two *Cygb* genes, NCBI Gene IDs 137366896 and 137359322, which encode identical proteins. The 137366896 gene is in a contig without flanking genes, whereas the 137359322 gene is in the expected genomic location. As with the *GbX* duplicates of the Cloudy catshark, we assumed that these genes are alleles rather than duplicates; hence, we retained the sequence of the 137359322 gene for all subsequent analyses. As in a previous study (Opazo et al. 2015a), we did not find matches for two elephant fish transcripts identified in a Sanger-sequencing study of full-length cDNAs (Tan et al. 2012). For the sake of consistency, these genes were not included in the counts, but they are included in the phylogeny to enable comparisons with the previous study.

### Globin Repertoires

In our curated set, the number of globins per genome ranges from seven in the three representatives of Holocephali (elephant fish, rabbitfish, and ratfish) to 34 in the giant manta (Fig. 1). Variation in the number of globins per genome is largely driven by changes in the α- and β-globin gene subfamilies, which range from 1 to 20 and from 1 to 9 copies per genome, respectively. *Cygb* was absent in most cartilaginous fish, whereas the *Adgb*, *GbY*, *GbX*, and *Mb* genes were present as single copies in all taxa. As in our previous report (Opazo et al. 2015a), we did not find traces of either *Ngb* or *GbE* in any of the cartilaginous fish genomes we screened.

### Phylogenetic Analyses

**Fig. 2.**
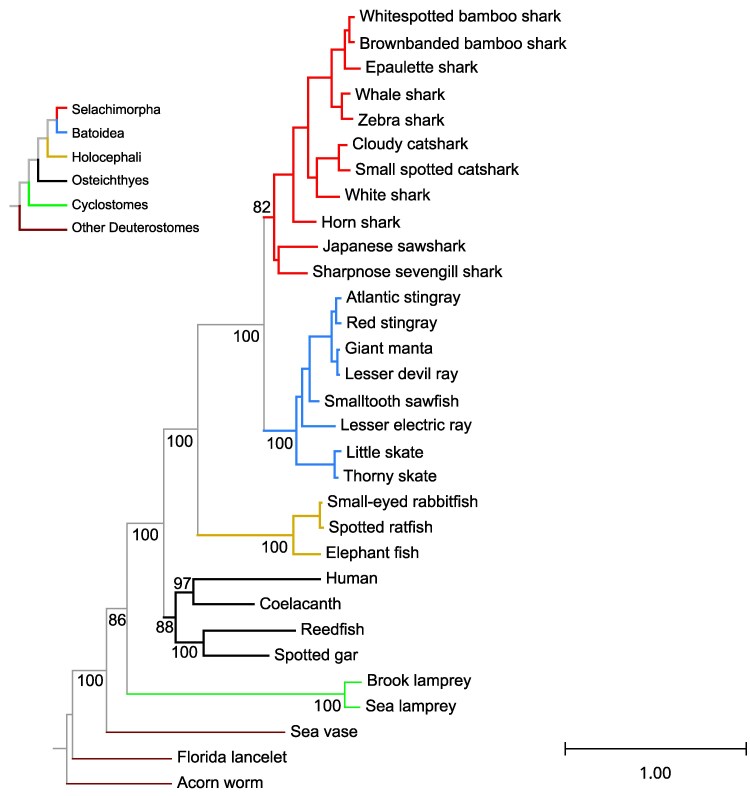
Maximum likelihood phylogram depicting relationships among the androglobin genes of cartilaginous fishes, with bony vertebrate and deuterostome sequences included for context. Numbers correspond to ultrafast bootstrap values. Shark branches are in red, batoid branches are in blue, and Holocephali branches are in fuchsia. The phylogeny was inferred under the Q.MAMMAL + I + G4 substitution model chosen by ModelFinder using a 2,319 aa alignment. The tree was rooted using the acorn worm androglobin sequence.

We conducted separate phylogenetic analyses for Adgb and all other globins combined. We separated Adgb from the other globins in our phylogenetic analysis because it is a much longer sequence, and there are non-globin domains present in the protein sequence. In addition, we conducted separate analyses for each globin subfamily where the arrangement of cartilaginous fish sequences deviated from the expected organismal relationships among Holocephali, Batoidea, and Selachimorpha. To provide evolutionary context, we included additional representatives of deuterostomes as well as bony vertebrates in all phylogenetic analyses. The estimated phylogeny of Adgb sequences (Fig. 2) matches the organismal tree (Fig. 1), with the Adgb proteins of Selachimorpha, Batoidea, and Holocephali forming reciprocally monophyletic groups, and Selachimorpha and Batoidea placed sister to one another.

**Fig. 3.**
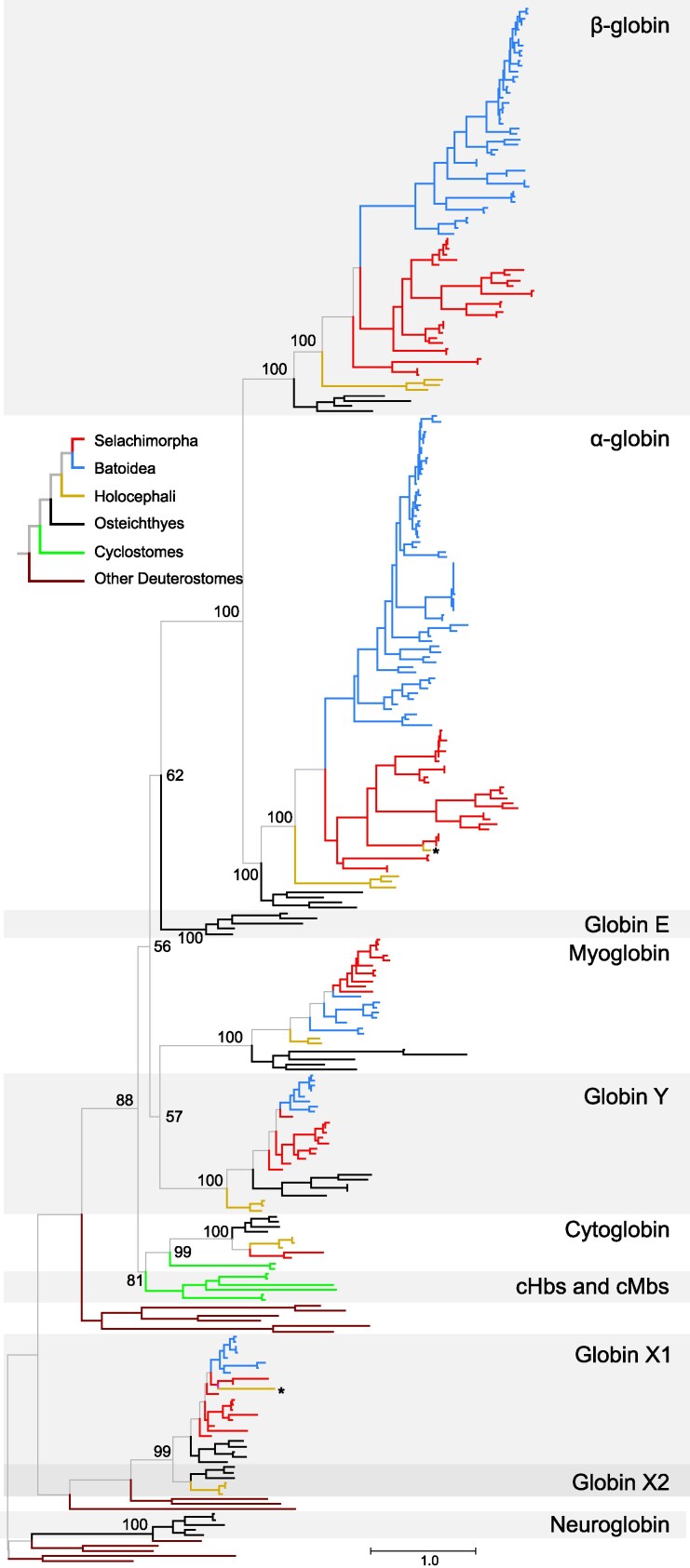
Maximum likelihood phylogram depicting relationships among the single-domain globin genes of cartilaginous fishes, with bony vertebrate and deuterostome sequences included for context. Numbers correspond to ultrafast bootstrap values. Shark branches are in red, batoid branches are in blue, and Holocephali branches are in fuchsia. Genes marked with asterisks are included in the phylogeny for comparative purposes, but they are not found in the current genome assemblies. The phylogeny was inferred under the JTT + R7 substitution model chosen by ModelFinder using a 314 aa alignment. The tree was rooted using the neuroglobin sequences. A version of this tree with terminal branches labeled is presented as Fig. S1, Supplementary material online).

In the case of all other globins other than Adgb, and in agreement with previous studies (Hoffmann et al. 2012a; Opazo et al. 2015a; Prothmann et al. 2020), the globins of vertebrates fell into three separate clades (Fig. 3, Fig. S1, Supplementary material online), with Ngb in one, GbX in the second, and vertebrate-specific globins in the third, with representatives of cartilaginous fishes in the latter two groups. The monophyly of the clade of vertebrate GbXs and of the clade of vertebrate-specific globins had high support, 99% and 88%, respectively, and the monophyly of each vertebrate specific globin subfamily had strong support as well, with bootstrap values of 99 or 100%, with the exception of GbX1, which had a bootstrap support value of 68% (Figs. 2 and 3, Fig. S1, Supplementary material online).

Within *GbXs,* we found *GbX1* copies in Selachimorpha and Batoidea, and *GbX2* copies in the Holocephali (Fig. 3, Fig. S1, and Table S1, Supplementary material online). As in a study by Opazo et al. 2015a, we failed to find the elephant fish *GbX1* gene in the current assembly and did not find any matches in the other two Holocephali either. Both GbX1 and GbX2 protein sequences are placed in monophyletic groups, with the sequences of bony vertebrates placed sister to those of cartilaginous fishes in both cases. In the case of GbX1, sequences from Batoidea were placed in a monophyletic group, whereas the GbX1 sequences from Selachimorpha were paraphyletic relative to the Batoidea. Interestingly, the elephant fish GbX1 is placed sister to the GbX1 sequences of catsharks (Fig. 3). Forcing the cartilaginous fish GbX1s of Batoidea and Selachimorpha to be monophyletic and the elephant fish GbX1 as sister to all other cartilaginous fish GbX1s did not result in a significant loss in likelihood score (Table S2, Fig. S2, Supplementary material online). We only found GbX2 copies in the Holocephali, and these sequences are placed in a monophyletic group sister to the GbX2s from bony vertebrates. Thus, the single-copy *GbX* genes of cartilaginous fishes represent a case of hidden paralogy (Kuraku 2010), resulting from reciprocal losses of alternative *GbX* ohnologs from a shared whole-genome duplication in vertebrates (Hoffmann et al. 2021). In the case of Holocephali, *GbX1* was retained, and in the case of Elasmobranchs, *GbX2* was retained. Synteny of the *GbX* ohnologs in cartilaginous fish compared to the spotted gar, a bony fish that has both *GbX* copies, supports the inference that the *GbX* ohnologs were differentially retained between the Holocephali and sharks, skates, and rays (Fig. S3, Supplementary material online). In the spotted gar, one *gbx* copy (*gbx1*) has shared synteny with the *GbXs* of sharks, skates, and rays, as all of these genes are in close proximity to *Spint2* and *Pleckhg2*. The other *gbx* copy of spotted gar has shared synteny with *GbX2* of the elephant fish, and no *GbX* genes were found in this region of shark, skate, or ray genomes.

Within vertebrate-specific globins, the α- and β-globin genes of jawed vertebrates are in sister monophyletic groups (Fig. 3, Fig. S1, Supplementary material online). Osteichthyes GbE is placed sister to the α- and β-globin clade, and this clade is sister to the group that unites Mb and GbY with the clade that includes Cygb and the cHbs and cMbs of cyclostomes at the deepest split of vertebrate-specific globins (Fig. 3, Fig. S1, Supplementary material online). Support for the nodes resolving relationships among the different subfamilies of vertebrate-specific globins is relatively low, ranging from 57% to 62%, with the exception of the sister relationship between the α- and β-globins of jawed vertebrates (bootstrap support of 100%), and the sister relationship between Cygb and the clade that unites cHbs and cMbs (bootstrap support of 81%). Within these paralogs, cartilaginous fish sequences fell in monophyletic groups in the cases of α- and β-globin, Cygb, and Mb, and the sequences for all three of Batoidea, Selachimorpha, and Holocephali fell in monophyletic groups in the α-globin subtree. The Holocephali paralogs always fell in monophyletic groups, whereas the Batoidea and Selachimorpha sequences did so in most cases. As in the case of GbX, we ran separate searches for the β-globin, GbY, and Mb paralogs to test whether forcing cartilaginous fish sequences to be monophyletic and, within them, forcing each of Holocephali, Batoidea, and Selachimorpha sequences to be monophyletic as well. Topology tests indicate that GbY was the only case where the constrained tree was significantly different from the maximum likelihood gene tree (Table S2, Figs. S4 to S6, Supplementary material online). A strict reconciliation of the GbY tree with the organismal tree would imply that many of these separate GbY clades derive from different ancestral genes. Since *GbY* has only been found as a single-copy gene when present, we infer that these genes are orthologs and attribute this unexpected phylogenetic arrangement to an unusual accumulation of substitutions in the stem of the GbY tree, as it minimizes duplication events. Two additional sources of evidence support our interpretation. Synteny of *GbY* is conserved across the major cartilaginous fish lineages, which supports our conclusion that there is not a complicated history of *GbY* duplication and loss (Fig. S7, Supplementary material online). In addition, an ultrametric version of the GbY tree was significantly worse than the maximum likelihood tree in a likelihood ratio test (*P* < 2e10^−101^), indicating that there are significant differences in evolutionary rates among the sequences.

To evaluate this further, we conducted codon-based branch model analyses (aBSREL) to test for differences in selection pressures along ancestral branches (Smith et al. 2015); first, we conducted an exploratory analysis using all branches to test for signatures of episodic diversifying selection, but we found no significant evidence of diversifying selection in any branch after aBSREL's *P-value* correction for multiple testing. Because exploratory analyses in aBSREL can be less sensitive to detect diversifying selection, we also tested for episodic diversifying selection along four specific branches of the *GbY* tree. The four branches are as follows: (1) the branch leading to the common ancestor of all Chondrichthyes, (2) the branch leading to the common ancestor of the Holocephali, (3) the branch leading to the common ancestor of the Selachimorpha, and (4) the branch leading to the common ancestor of the Batoidea. Of these four hypotheses, we detected evidence of episodic diversifying selection only in the branch leading to the Selachimorpha (Test LRT number = 6.5, *P*-value = 0.013). This could partially explain the discordance between the *GbY* tree and the expected organismal phylogeny. The alignment and phylogeny used for aBSREL analyses are provided in the supplementary materials online, and the full table of aBSREL results is provided in Table S3, Supplementary material online.

As for most other vertebrate groups, the only globin paralogs to exhibit large differences in gene copy numbers are the α- and β-globins. The three Holocephali species have a single copy of each gene, grouped in strongly supported monophyletic clades that are placed sister to the elasmobranch sequences. Within elasmobranchs, sharks and batoids exhibit contrasting patterns of evolution in the α- and β-globins. The α- and β-globin genes of sharks are arranged in species-specific clades, which suggests either a rapid rate of gene turnover or gene conversion. In the case of gene turnover, each lineage would have begun with a single copy of α- and β-globin, followed by species-specific tandem duplications and losses following the “gene birth and death” model of evolution (Ota and Nei 1994). In the case of gene conversion, a globin paralog will non-reciprocally recombine with a neighboring paralog, eventually making sequences in a tandem cluster homogenous in a process called concerted evolution (Liao 1999). There is evidence of α- and β-globins evolving via rapid gene turnover and concerted evolution (Erhart et al. 1985, 1987; Storz et al. 2007; Hoffmann et al. 2008a, 2008b, 2018; Opazo et al. 2015b; Storz 2019; Signore et al. 2019; Desvignes et al. 2025). Batoid duplicates exhibit a more complex pattern, with both species-specific expansions, such as a 10-gene expansion of α-globin genes in the giant manta, and multiple α- and β-globin duplications shared between the giant manta and the lesser devil ray (Fig. S1, Supplementary material online).

### Synteny Analyses

In most cartilaginous fish, *Adgb* is flanked by *Stxbp5* and *Sash1a* on one side, and by *Rab32* and *Grm1* on the other side (Fig. S8, Supplementary material online). This arrangement is shared with humans, spotted gar, and lamprey. *Mb* synteny is less well-conserved. In the elephant fish, *Mb* is flanked by *Foxred2* and *Luc7l2* on one side and *Smdt1b* and *Ndufa6* on the other. In sharks and batoids, it is flanked by *Naga* and *Serhl* on one side and by *Luc7l2* and *Ddx59* on the other. In spotted gar, the *Serhl* ortholog flanks *Mb*, but this arrangement is not shared with humans (Fig. S9, Supplementary material online).

In elephant fish, *Cygb* is flanked by *Qrich2* and *Rhbdf2* upstream and by *Prpsap1* and *Rnf157* downstream (Fig. S10, Supplementary Materials online). *Cygb* is flanked by *Prpsap1*, *Qrich2*, and *Rnf157* downstream in the horn shark and white shark, and by *Ube2o* and *Aanat* upstream in the white shark (Fig. S10, Supplementary material online). This information is lacking for the horn shark *Cygb*, as the gene is at the beginning of a scaffold. In human and spotted gar, the orthologs of *PRPSAP1*, *RNF157*, *QRICH2*, *RHBDF2*, *UBE2O*, and *AANAT* are all very close to the *Cygb* gene, suggesting that when present, the *Cygb* genes of cartilaginous fishes or their traces are in the ancestral location (Fig. S10, Supplementary material online).

The *GbY* gene is located at one end of the cluster of the α- and β-globin genes. The *α-*, *β-*, and *GbY* globin cluster is flanked by *Aanat2* and *Rhbdf1* on one end, and by *Luc7 l* on the other end in the vast majority of cartilaginous fishes. Unlike most other vertebrate lineages, *Nprl3*, a gene which is normally located immediately adjacent to the multigene hemoglobin clusters of lampreys and bony vertebrates and contains cis-acting regulatory elements in its introns (Higgs et al. 1990; Hay et al. 2016; Miyata et al. 2020), is found on a separate chromosome (Fig. S11, S12, Supplementary material online). In mammals, the regulatory elements found within *Nprl3* drive developmental changes in the expression of the α-globin genes in the adjacent cluster in the precursors of red blood cells (Miyata et al. 2020; Preston et al. 2025). The basic unit of the *Hb* clusters of cartilaginous fishes is a co-linear pair of α- and β-globin genes, as in the elephant fish and the whale shark. Expansions of the cluster are the consequence of the duplication of this basic unit in an anti-parallel orientation, as seen in Japanese sawshark, zebra, epaulette, and sharpnose sevengill shark, as well as the smalltooth sawfish. Additional expansions following the same pattern are seen in the more complex clusters of the lesser devil ray and Atlantic stingray (Fig. S11b, Supplementary material online).

### Structural Considerations of α- and β-globins

**Fig. 4.**
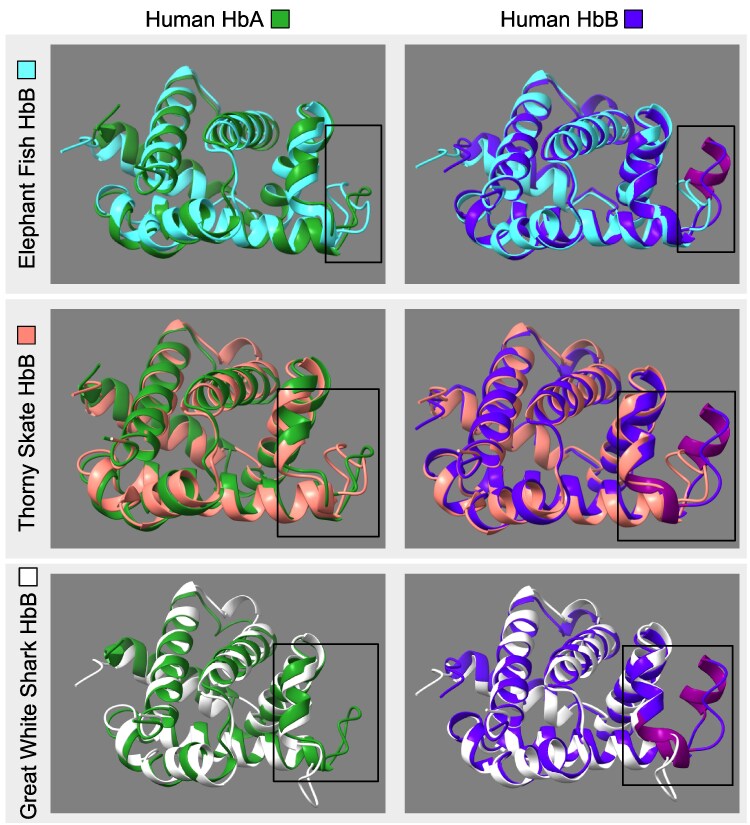
Structural comparison of human and cartilaginous fish Hb AlphaFold models. The predicted structures of α- and β-globin subunits from cartilaginous fish on top of human α- and β- subunits. The boxed area highlights the helix on the human β- globin subunits which is not found on the β-globin subunits from cartilaginous fishes. Accession numbers are as follows: P69905 and P68871 for human α- and β-globin, for elephant fish β-globin: NP_00127970, thorny skate β-globin: XP_032896874, and great white shark β-globin: XP_041061644.

The β-chain *Hbs* of sharks and batoids differ from the globins of most vertebrates in lacking a portion of the D-helix (Fisher et al. 1977; Chong et al. 1999; Naoi et al. 2001; Verde et al. 2005). A visual inspection of the β-globin alignment revealed that all cartilaginous fish sequences share the deletion of the D helix, positions 54 to 57 in the β-globin alignment or 164 to 167 in the full alignment, and that Orectolobiform and Carcharhiniform sharks have a second deletion in the same region that involves the first three amino acids of the E Helix, positions 58 to 60 in the β-globin alignment or 168 to 171 in the full alignment. The first of these deletions maps to the stem of the tree of cartilaginous fishes, and the second one to the last common ancestor of Orectolobiform and Carcharhiniform sharks. Vertebrate α-globins have a deletion in the same location, and structural predictions suggest that the β-globins of cartilaginous fish are structurally more similar to human α-globin than to human β-globin (Fig. 4). The D helix appears to be present in all other sequences in our full alignment, except for two GbE proteins from lungfish and reedfish. Thus, our results indicate that the α-globins of all jawed vertebrates, the β-globins of cartilaginous fish, and the two GbE listed above convergently lost this helix. The functional relevance of these deletions is unclear, as deleting the D helix in the β-globin chains of human hemoglobins has minimal impact on the oxygen binding affinity of the tetramer (Komiyama et al. 1991).

### Gene Expression

**Fig. 5.**
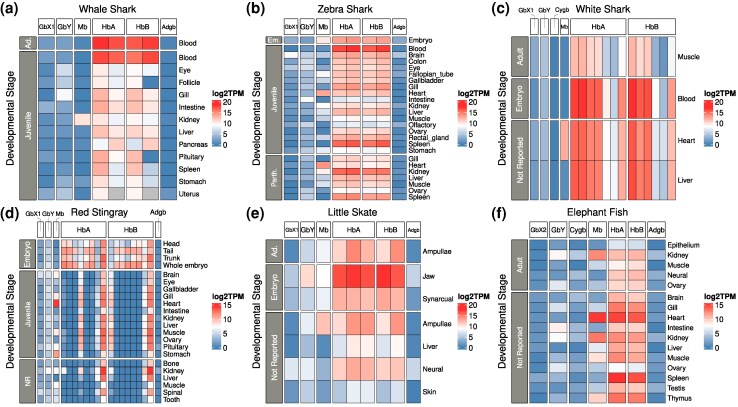
Expression of cartilaginous fish globin genes across tissues and developmental stages. Expression estimates were derived from log2-transformed TPM values assigned by Kallisto. Tissues and developmental stages were labeled according to information in the SRA run selector metadata. The full selection of taxa is shown in Fig. S13, Supplementary material online. Parth., Parthenote; Em., Embryo; Ad., Adult; NR, Not Reported.

We screened 689 RNA-seq libraries from cartilaginous fish to assess the expression profiles of the different globin paralogs. As expected, we found that the *Hb* and, to a lesser extent, *Mb* genes are the most highly expressed in all species (Fig. 5, Fig. S13, Supplementary material online). Myoglobin is most highly expressed in the heart, whereas the α- and β-globin genes are most highly expressed in blood. While *Adgb* was expressed in the brain, kidney, and various embryonic tissues, it was also expressed in the testis (when testis data were present), consistent with observations in mammals (Hoogewijs et al. 2012; Keppner et al. 2022). *GbX1* was broadly expressed, with the highest expression in neural tissues and the eye, consistent with previous findings (Fuchs et al. 2006; Opazo et al. 2015a; Gallagher and Macqueen 2017; Hoffmann et al. 2021). On the other hand, we only observed low-level expression of *GbX2* in a few tissues, suggesting that the role of *GbX* paralogs may not be conserved across cartilaginous fish lineages. *GbY* is expressed across many tissues; however, we observed the highest expression in the skin, brain, gill, spleen, kidney, and intestine for the various taxa used in our study. This is consistent with previous studies (Schwarze et al. 2015; Opazo et al. 2015a; Gallagher and Macqueen 2017), and the function of GbY remains unclear (Keppner et al. 2020). *Cygb* was only present in a few species—the horn shark, the elephant fish, and the white shark. Of these, the elephant fish has low levels of *Cygb* expression in the kidney, neural tissues, and reproductive tissues, whereas the *Cygbs* of the horn shark and white shark are not expressed in any of the tissues we measured (Fig. 5, Fig. S13, Supplementary material online). Interestingly, the Cygb sequences of the horn shark and white shark lack premature stop codons. They share a small deletion at positions 19 to 20 of the alignment (see Supplementary Materials online), and the white shark Cygb is missing seven amino acid positions at its terminal end, and it is more divergent than the other Cygbs included in this study, as shown by its long branch length (Fig. 3, Fig. S1, Supplementary material online).

We see different patterns of expression in species with multiple α- and β-globin paralogs. In the red stingray, most α- and β-globin paralogs are highly expressed in early development (embryonic tissues), but the number of expressed paralogs diminishes in later developmental stages (Fig. 5d). Expression data from the giant manta and the Atlantic stingray is consistent with this: a limited number of their α- and β-globin paralogs appear to be responsible for providing α- and β-globin chains for hemoglobin assembly (Fig. S13j, k, Supplementary material online). However, RNA-seq data from embryonic tissues in these species are lacking to confirm that more paralogs are expressed in earlier developmental stages. This does not seem to be the case in the white shark, where the proportional contribution of the different α- and β-globin paralogs does not seem to change much between embryos and adults (Fig. 5c). Finally, in most species with less than 3 *Hb* paralogs per subfamily, all paralogs seem to be expressed, with small differences among them (Fig. S13, Supplementary material online). The exception here is the lesser electric ray, where one of the three α-globin paralogs is expressed at much lower levels than the other two, and the whale shark, where one of two β-globin paralogs is expressed at much lower levels in follicle and pituitary tissues (Fig. S13n, Supplementary material online). As in teleost fish, there does not seem to be a correlation between position in the Hb cluster and timing of expression (Opazo et al. 2013). The functional relevance of the observed expansions of the α- and β-globins of cartilaginous fishes is not clear. There is limited information on their hemoglobins. The few studies available indicate that when present, the different isoforms are functionally very similar (Russo et al. 2017; Verde et al. 2005; Weber et al. 1983).

### Ancestral Reconstruction and Evolution of the Globin Repertoire

The *Adgb* gene tree is concordant with the known organismal phylogeny, suggesting that this gene was present in the last common ancestor of cartilaginous fishes as a single copy and has remained in this state in all descendant lineages. A strict reconciliation of the subtrees of the different globin paralogs in Fig. 3 with the organismal tree in Fig. 1 is straightforward for both α-globin and *Cygb* and implies that these genes were present in a single copy state in both the last common ancestor of cartilaginous fishes and the last common ancestor of elasmobranchs and that *Cygb* was secondarily lost multiple times independently (Fig. 1). The cases of *Ngb* and *GbE* are also simple. Since they are absent from all the genomes screened, the most parsimonious inference is that they were also absent from their last common ancestor. This strict reconciliation would also imply the presence of multiple *GbX1s* and *GbYs* in the last common ancestor of cartilaginous fishes, and of multiple *GbX1s*, *GbYs*, *Mbs*, and β-globins in the last common ancestor of elasmobranchs. However, topology tests cannot distinguish between the best subtrees and those that forced Batoidea, Selachimorpha, and Holocephali sequences into monophyletic groups. Thus, we infer that these genes were also in a single copy state in the common ancestor of cartilaginous fishes and the common ancestor of elasmobranchs. In the case of *GbY*, as explained above, we also selected the constrained tree because we suspect that this result is a combination of unusually high rates of evolution in the bony fish portion of the tree and annotation inconsistencies.

Ignoring the elephant fish globins that were not present in the genome assemblies, the globin repertoire of the last common ancestor of cartilaginous fishes is inferred to have had single copies of *Adgb*, α- and β-globin, *Cygb*, *Mb*, *GbY*, *GbX1,* and *GbX2*. This is consistent with what was initially observed in the elephant fish (Opazo et al. 2015a) and expands that work using a large sampling of cartilaginous fishes. From the ancestral state, the only change in the branch leading to the last common ancestor of Holocephali is the loss of *GbX1*. Our study is the first to investigate the globin repertoire in the elasmobranchs, the other side of the cartilaginous fish phylogeny. The only change in the branch leading from the last common ancestor of cartilaginous fishes to the last common ancestor of elasmobranchs is the loss of *GbX2*, along with the loss of *Cygb* in the last common ancestor of batoids, as well as in three different lineages in the Selachimorpha. The α- and β-globin subfamilies expanded independently in Selachimorpha and Batoidea. In the case of Selachimorpha, all these expansions lead to species-specific clades, whereas in the case of Batoidea, there are both species-specific expansions as well as older ones shared among different species. These expansions were most prominent in the α-globin gene family relative to the β-globin in most species, with the giant manta providing the most extreme case with 20 copies of α-globin and 9 of β-globin. The number of ancestral globin genes is summarized in Table S4 (Supplementary Materials online).

### Evolution of the Globin Superfamily in Cartilaginous Fish and Bony Vertebrates

The globin repertoires of cartilaginous fish differ in key aspects relative to those of bony vertebrates. Cartilaginous fish lack *Ngb*, a globin that was present in the common ancestor of vertebrates and is present in almost all bony vertebrates and is highly expressed in the brain and other neural tissues (Burmester et al. 2000; Dröge et al. 2012). *Cygb* is another highly conserved globin in bony vertebrates that, in cartilaginous fish, is either entirely absent, nonfunctional, or expressed at very low levels. By contrast, all surveyed cartilaginous fish retain a single copy of *GbY*, a gene that has been lost multiple times in bony vertebrates (Storz et al. 2011; Schwarze et al. 2015; Hoffmann et al. 2018).

Another notable difference between cartilaginous fish and bony vertebrates is the translocation of *Nprl3* from its ancestral chromosomal location upstream of the α- and β-globin clusters and *GbY* (Figs. S11, S12, Supplementary material online). This opens interesting questions regarding the regulation of *Hb* expression in cartilaginous fish because ancient regulatory elements in the introns of *Nprl3* are linked to the regulation of where and when the different *Hb* genes of cyclostomes and gnathostomes are expressed (Higgs et al. 1990; Hay et al. 2016; Miyata et al. 2020), although these hemoglobins have independent evolutionary origins (Hoffmann et al. 2010). Either cartilaginous fishes have acquired novel regulatory elements, as with the translocated β-globin gene clusters of amniotes, or the same elements can act in trans conformation.

As for the *Hb* clusters themselves, it is interesting to note that tandemly arranged clusters of *Hb* genes seem to have expanded independently from a single gene multiple times in the evolution of jawed vertebrates (Opazo et al. 2008, 2013, 2015b; Hoffmann et al. 2018): in the expansions of *Hb* clusters of sharks (1), of batoids (2), and the β-globin cluster of amniotes (3). In most bony vertebrates, these expansions of the hemoglobin clusters fueled the emergence of hemoglobins with specialized biochemical roles, such as the Root-effect hemoglobins of teleost fish, which enhance oxygen delivery to tissues such as the swim bladder and the retina (Root 1931; Root and Irving 1943; Rummer and Brauner 2015), and the embryonic and fetal hemoglobins of mammals (Fantoni et al. 1981). The hemoglobin and myoglobin genes of lampreys are also arranged in clusters that are independent in origin relative to the hemoglobin clusters of jawed vertebrates (Schwarze et al. 2014) and also evolved specialized functional and developmental roles (Fago et al. 2018). Our analyses suggest that a similar pattern is present in both sharks and batoids, as there are differences in the time and tissues where the different hemoglobin genes are expressed (Fig. 5, Fig. S13, Supplementary material online).

In conclusion, our study shows that the early evolution of the globin gene superfamily of cartilaginous fishes involved the loss of several paralogs present in the last common ancestor of jawed vertebrates, and the differential retention of some paralogs relative to bony vertebrates. Such patterns suggest that the division of labor among globin paralogs may have evolved differently in each of the two main lineages of jawed vertebrates. Finally, our study is the first to note that *GbX* paralogs were differentially retained in the major cartilaginous fish lineages.

## Materials and Methods

### Bioinformatic Searches

We screened the genomes of 20 cartilaginous fish species with genomes annotated using the NCBI RefSeq eukaryotic genome annotation pipeline (last accessed April 30th 2025). This sample includes 11 shark species covering 5 of the 8 orders in the Selachimorpha, 8 batoid species including 4 species in order Myliobatiformes, a sawfish (order Rhinopristiformes), an electric ray (order Torpediniformes), and 2 skates (order Rajiformes), and a sole representative of the order Holocephali (Table 1, Table S1, Supplementary material online). Ref-seq genomes and their annotations (in GFF3 format) were downloaded from NCBI using BiomartR (Drost and Paszkowski 2017). To increase representation of the Holocephali, we manually annotated the globin repertoire of a ratfish and a rabbitfish, two additional species in the group for which there are reference genomes available that do not meet the Ref-seq criteria.

To search for globin sequences, we combined searches on protein databases seeded with the amino acid sequences using blastp, searches of translated nucleotide databases seeded with amino acid sequences using tblastn, and searches of nucleotide databases seeded with nucleotide sequences using blastn. Initial searches were seeded with a representative set of known globins from deuterostomes that included the full repertoire of globins of the elephant fish as reported in a previous study by Opazo et al. 2015a, and added the Adgb, α-globin, β-globin, Cygb, Mb, and Ngb protein sequences of humans, plus the GbE, GbX, and GbY proteins from coelacanth. First, a protein blast database was made using publicly available gene models built by NCBI of all cartilaginous fish of interest. Next, globin seeds were aligned to the database using blastp with an e-value cutoff of 1e^−5^. Summary information was downloaded for the resulting hits using NCBI datasets v17.3.0 (O’Leary et al. 2024). NCBI GeneIDs were obtained for each protein, and only the longest isoform of each gene was used for a subsequent phylogenetic analysis to confirm their homology to the original globin seeds. To do this, the longest protein isoforms were aligned along with the globin search seeds using mafft's (v7.490) l-ins-i algorithm (Katoh and Standley 2013), and a phylogenetic tree was inferred using IQ-Tree v2.0.7 under the JTT + F + R6 model (Nguyen et al. 2015; Minh et al. 2020). Support for the nodes was evaluated with 10,000 ultrafast bootstrap replicates (Hoang et al. 2018). This tree was only used to confirm homology among our initial searches, and a more comprehensive sampling was used for our final phylogenetic analysis (see subsequent methods). A representative set of the globins from cartilaginous fishes was added as seeds when searching for globins that were not found in our initial search.

### Data Curation

Because of differences in the protein domain structure, bioinformatic searches and phylogenetic analyses were conducted separately for Adgb, which includes a globin domain with the helices rearranged and fused to additional protein domains, and for all other globins, which include a single globin domain, with some terminal extensions. In the case of searches seeded with Adgb, we discarded sequences that only matched the calpain domain, which were retrieved in our initial searches.

We used similarity scores for the initial classification of the sequences into the different globin types. These initial classifications were adjusted with the results of phylogenetic and synteny analyses. Almost all genomes possess copies of *Adgb*, *GbY*, and *Mb* identified in the annotated genes through our blastp searches. Whenever these genes were absent from an assembly, we used alternative approaches to get the sequences. In the case of *Adgb*, because of the complexity of the gene, we only used tblastn searches, ordering the hits by position along the query, and patching the fragments, removing spurious overlaps. Although the *Adgbs* of white shark, ratfish, and rabbitfish were found in the corresponding genomes, the hits cover >75% of the translated protein and are clearly incomplete. We infer that these genes are present and functional, but not fully covered in the current genome releases. We included the corresponding partial sequences in our phylogenetic analyses, but the resulting gene models need to be verified with additional data. In the case of *Cygb*, *GbY*, and *Mb*, we combined tblastn to identify the fragment where the gene was located, with blastn searches seeded with the *Cygb*, *GbY*, or *Mb* exons from a closely related cartilaginous fish to manually annotate the genes. When we searched the nucleotide databases using globin exons as seeds, we identified the first exon of *GbY* in the lesser electric ray genome but found no trace of exons 2 or 3. Because *GbY* is present in all other cartilaginous fishes, we surveyed transcriptome data to look for this gene. Doing so, we were able to obtain the full coding sequence of this *GbY* gene by assembling de novo a transcriptome from the lesser electric ray and matching transcripts to this exon (see Supplementary Information, Supplementary material Online). We failed to find any matches that would correspond to exons 2 or 3 in the RefSeq genome; hence, we assume this gene is present in this species but absent from the current assembly. Exons 2 and 3 from the red stingray *GbY* gene are annotated as part of the *Luc7 l* gene in the current annotation, GeneID 140735596, so we reannotated the first exon by comparison with the *GbY* exons of the closely related Atlantic stingray. Using a similar approach, we found and exon of the *Mb* gene of the ratfish, but because there are no RNA-Seq data sets available for the ratfish, we were unable to obtain the full coding sequence for the *Mb* gene. We assume that the *Mb* gene is present in the ratfish but is simply missing from the current assembly. Because of the short length of the fragment, we did not include the sequence in the phylogenetic analysis. Finally, in the case of *GbX*, we also used synteny information to classify the genes into either *GbX1* or *GbX2*, following Gallagher and Macqueen (Gallagher and Macqueen 2017), as we did in (Hoffmann et al. 2021).

We found four genes annotated as globins of unusual lengths, such as α- and β-globins longer than 200 amino acids. These genes were blasted back to the cartilaginous fish protein database, excluding the source species. In the case of the 116985437 and 109935067 genes of thorny skate and whale shark, respectively, both are α-globins fused with a β-globin that were annotated as separate genes in previous genome releases; thus, the older annotations were used: ENSARAG00005009971 and ENSARAG00005009980 for the thorny skate and ENSRTYG00015001848 and ENSRTYG00015001843 for the whale shark. In addition, the 121288390 gene of the white shark and the 122539636 gene of the white-spotted bamboo shark are also longer than our threshold. In these cases, the genes were discarded because they included non-globin-like domains.

### Phylogenetic Analyses

For all phylogenetic analyses, we aligned the predicted amino acid sequences using the L-INS-i strategy from Mafft v7.505 (Katoh and Toh 2008; Katoh and Standley 2013). We then estimated phylogenetic relationships among the sequences using IQ-Tree version 3.0.1 (Wong et al. 2025), using the ModelFinder routine to estimate the best-fitting model of amino acid substitution (Kalyaanamoorthy et al. 2017), selecting the model chosen by the Bayesian Information Criterion, and using the resulting models in the searches (see log file in Supplementary Materials online). We evaluated support for the nodes with 10,000 replicates of the ultrafast bootstrap routine (Hoang et al. 2018). We compared competing phylogenetic hypotheses using constrained searches and the approximately unbiased topology test proposed by Shimodaira (Shimodaira 2002) as implemented in IQ-Tree. A full description of the commands needed to replicate the analyses is available in Supplementary File S1, Supplementary material online.

### Selection Analysis

We collected the coding sequences for the longest *GbY* isoforms using NCBI datasets. When a sequence was not annotated in NCBI, we collected the coding sequences from our previous manual annotations. We created a codon-based alignment using Aliview's “align as translated amino acid” tool (Larsson 2014). We appended the previously generated constrained phylogeny (Fig. S5, Supplementary material online) to the end of the alignment file and used the resulting file as input for the aBSREL (Smith et al. 2015) test via HyPhy v2.5.2 (Kosakovsky Pond et al. 2020). All branches were tested for signatures of selection. The input file used for aBSREL analysis (alignment + constrained GbY phylogeny) is provided in the Supplementary material online.

### Synteny Analyses

We used a custom bash script to parse GFF annotations of the taxa used in this study and obtain genes flanking globins. Next, the longest protein isoforms corresponding to these genes were downloaded using NCBI datasets v17.3.0 (O’Leary et al. 2024). These proteins were aligned to the human reference proteome using blastp and were labeled with the gene name of the closest human blast hit. Syntenic regions were visualized using the gggenomes v1.0.1 package in R v4.4.2 (R Core Team 2020; Hackl et al. 2024).

### Organismal Phylogeny and Divergence Dates

Phylogenetic relationships among cartilaginous fish species are based on the results of molecular studies (Aschliman et al. 2012; Naylor et al. 2012), and divergence times among the different groups correspond to the median time estimates provided by TimeTree v5 (Kumar et al. 2022).

### Structure Analyses

We investigated the structural differences between the α- and β-hemoglobin subunits of three cartilaginous fish, a great white shark (Selachimorpha), thorny skate (Batoidea), and elephant fish (Holocephali), and humans using the publicly available AlphaFold (Accessed May 1st, 2025). The protein sequences were uploaded to the web server, and a predictive 3D model was made from each of the protein sequences. The models were downloaded, and the CIF files were imported into ChimeraX v1.9 (Meng et al. 2023), where two of these models were overlayed on top of each other using the alignment feature.

### Gene Expression Analysis

RNA-seq datasets from the cartilaginous fish used in our study were downloaded from the Sequence Read Archive (SRA) using the search term provided in the Supplementary Information, Supplementary material online. Metadata for the resulting datasets was obtained from the SRA run selector (Table S5, Supplementary material Online). Sequence data was downloaded from the SRA using the SRA toolkit commands “prefetch” and “fasterq-dump” (Leinonen et al. 2010).

Reference transcriptomes for each species used in our study were downloaded from NCBI using datasets (O’Leary et al. 2024). Separate Kallisto (v0.51.1) indices were made from each dataset using kallisto index, and reads were pseudo-aligned separately to the transcriptome indices using kallisto quant (Bray et al. 2016). Globin transcripts were extracted from Kallisto's transcript abundance results and were labeled with their corresponding SRA datasets, GeneIDs, tissues, and developmental stages. Expression values for globin genes were derived from the sum transcripts per million (TPM) over all isoforms and log2 transformed for visualization. Manually annotated globin transcripts were added to their species’ transcriptome before indexing. Expression was visualized using the ComplexHeatmap v2.22.0 package in R (Gu et al. 2016; R Core Team 2020).

## Supplementary Material

evag058_Supplementary_Data

## Data Availability

All data used in this study were previously generated and can be found in NCBI's RefSeq, GenBank, or sequence read archive (SRA) databases. Accession numbers for genomes used in this study are provided in Table 1. Metadata associated with globin sequences derived from bioinformatic searches is shown in Table S1. Metadata related to SRA datasets used in the gene expression portion of this study are provided in Table S5. Amino acid alignments, phylogenetic trees, a single file containing the phylogeny and codon alignment for aBSREL analysis, and a log file to reproduce the phylogenetic analysis are provided in the Supplementary Materials online.
