# Supplementary material for: A Deep Dive into the Globin Superfamily of Sharks, Skates, and Rays: Contrasting Patterns of Gene Loss and Retention Relative to Bony Vertebrates
Source: Genome Biol Evol. 2026 Mar 13;18(3):evag058. doi: 10.1093/gbe/evag058 (PMC13023373; doi:10.1093/gbe/evag058)
Supplement: evag058_Supplementary_Data [file evag058_supplementary_data.zip › Supplementary_tables_and_figures_R3.pdf]

Supplementary Information:

Search term for NCBI SRA (Conducted on May 7, 2025)

(("Chiloscyllium plagiosum"[Organism] OR Chiloscyllium plagiosum[All Fields]) OR ("Pristis pectinata"[Organism] OR Pristis pectinata[All Fields])) OR Amblyraja radiata OR Carcharodon carcharias OR Callorhinchus milii OR Hemiscyllium ocellatum OR Rhincodon typus OR Leucoraja erinacea OR Hypanus sabinus OR Stegostoma tigrinum OR Heptranchias perlo OR Narcine bancroftii OR Pristiophorus japonicus OR Scyliorhinus canicula OR Mobula hypostoma OR "Scyliorhinus torazame"[Organism] OR "Scyliorhinus torazame"[All Fields]) OR ("Mobula birostris"[Organism] OR "Mobula birostris"[All Fields]) OR ("Hemitrygon akajei"[Organism] OR "Hemitrygon akajei"[All Fields]) OR ("Chiloscyllium punctatum"[Organism] OR "Chiloscyllium punctatum"[All Fields]AND ("biomol rna"[Properties] AND "library layout paired"[Properties])

Datasets removed after search

SRR12550959 ignored because it was ncRNAseq

SRR21023205-SRR21023210 ignored because it was amplicon

SRR27125045-SRR27125050 ignored because it was Assay type "other" with no information.

SRR31783813-SRR31783816 ignored because it was Chipseq

Lesser Electric Ray Transcriptome Assembly

SRA datasets SRR29142957 and SRR29142959 derived from lesser electric ray (LER) tissues were concatenated together and read depth was normalized to a depth of 10,000 using Trinity's insilico_read_normalization.pl script (Grabherr et al., 2011). These reads were then provided to Trinity via their docker container with the –no_normalize_reads option on. The LER transcriptome was made into a nucleotide blast database, and a partial GbY sequence derived from the LER reference genome (exon 1 of the putative GbY) was alinged to the transcriptome using blastn. From the resulting blast hits, we found at transcript of length 1,520 nt, encoding for a 160 aa protein with high homology to other catilaginous fish GbYs.

Supplementary Table 2. Results of topology tests where we compared the maximum likelihood tree (ML) for each paralog with one where the Holocephali, Batoidea and Selachimorpha sequences were constrained to be monophyletic. All tests were run in IQ-Tree 3.0.1 for Linux 64-bit (Wong et al. 2025)

Globin paralog	logL of ML tree	logL of constrained tree	ΔL	p-AU
β-globin	-7094.6	-7095.4	0.8	0.28
Mb	-3734.7	-3748.5	12.1	0.14
GbY	-3181.8	-3191.1	9.3	0.03 *
GbX	-6250.3	-6255.1	2.0	0.34

ΔL: difference in likelihood score
p-AU: p-value of approximately unbiased (AU) test (Shimodaira, 2002)

References

Shimodaira H. 2002. An approximately unbiased test of phylogenetic tree selection. *Syst Biol*. 513:492–508.

T.K.F. Wong, N. Ly-Trong, H. Ren, H. Banos, A.J. Roger, E. Susko, C. Bielow, N. De Maio, N. Goldman, M.W. Hahn, G. Huttley, R. Lanfear, B.Q. Minh. 2025. IQ-TREE 3: Phylogenomic Inference Software using Complex Evolutionary Models. Submitted, https://doi.org/10.32942/X2P62N.

Supplementary Table 4. Inferred number of genes in the globin gene repertoire of ancestral cartilaginous fishes.

Ancestor	*Adgb*	Ngb	GbX1	GbX2	GbY	Cygb	GbE	Mb	α-Hb	β-Hb
Selachimorpha	1	0	1	0	1	1	0	1	1	1
Batoidea	1	0	1	0	1	0	0	1	1	1
Holocephali	1	0	0	1	1	1	0	1	1	1
Elasmobranchii	1	0	1	1	1	1	0	1	1	1
Chondrichthyes	1	0	1	1	1	1	0	1	1	1

Supplementary Figure 1

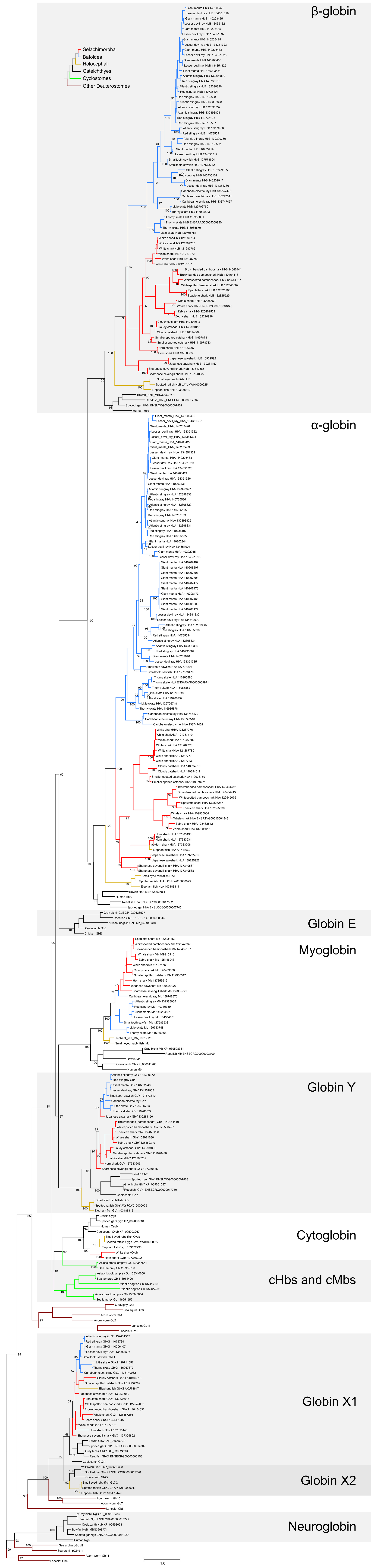

Supplementary Figure 1. Maximum likelihood phylogram depicting relationships among the single-domain globin genes of cartilaginous fishes, with bony vertebrate and deuterostome sequences included for context. Numbers correspond to ultrafast bootstrap values. Branches are colored according to the tree in the inset. Genes marked with asterisks are included in the phylogeny for comparative purposes but they are not found in the current genome. The tree was rooted using the neuroglobin clade.

Supplementary Figure 2

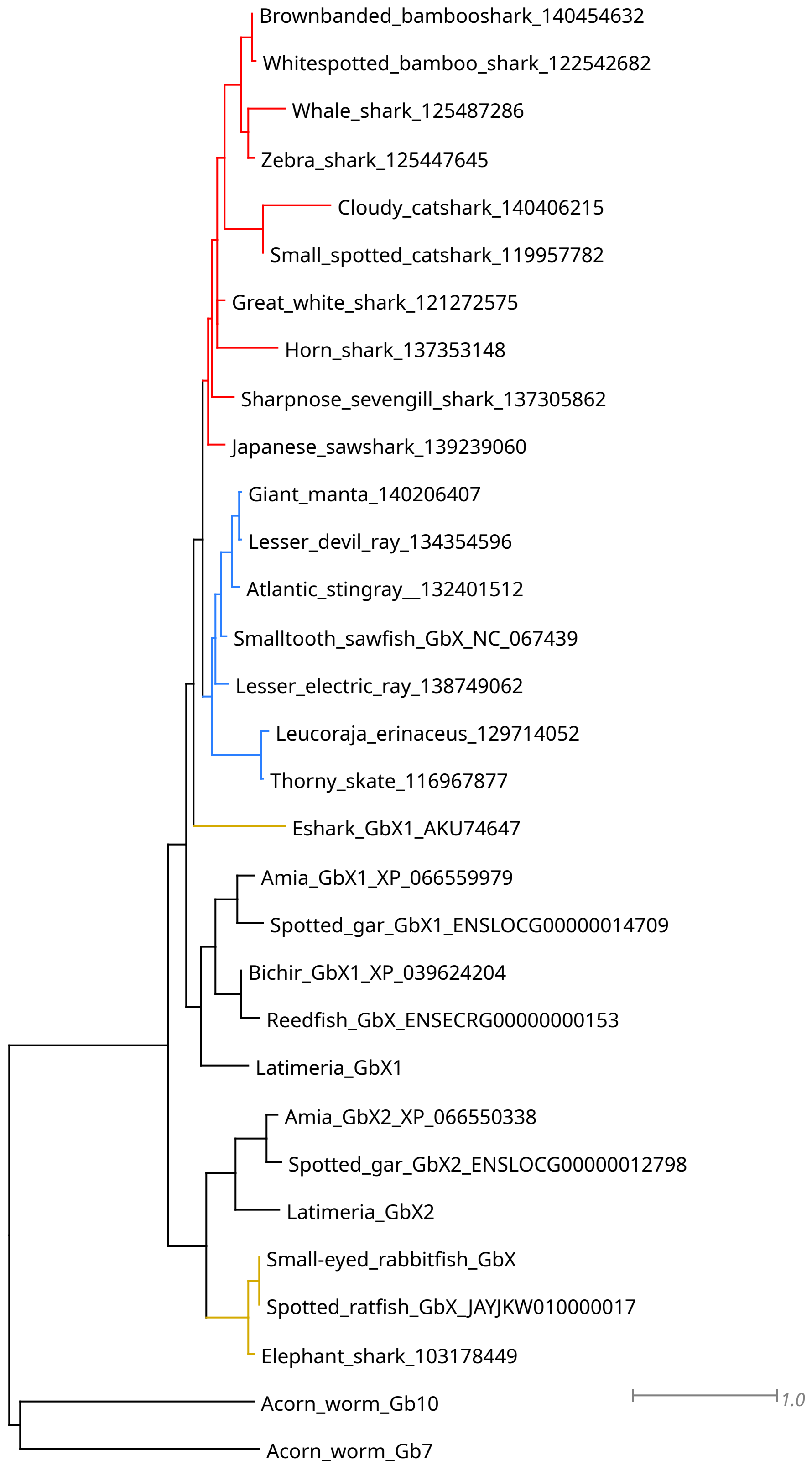

Supplementary Figure 2. Maximum likelihood phylogram depicting relationships among the GbX proteins of cartilaginous fishes forcing GbX1 and GbX2 to be reciprocally monophyletic while constraining the cartilaginous fish GbX1s of Batoidea and Selachimorpha to be monophyletic. The tree was rooted using acorn worm GbX sequences. Shark branches in red, batoid branches in blue and holocephali branches in fuchsia.

Supplementary Figure 3

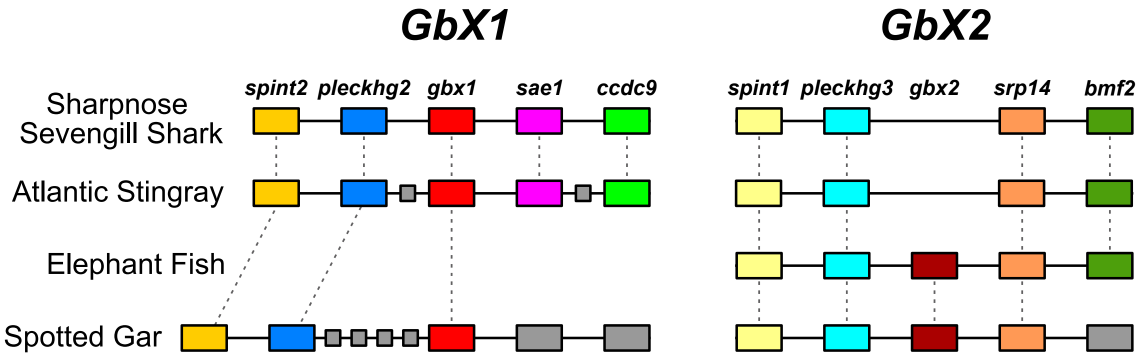

Supplementary Figure 3. *GbX* synteny reveals differential retention of *GbX* ohnologs after the shared vertebrate whole genome duplication. Four representative taxa from each major cartilaginous fish lineage is shown. The *GbX1* locus of the elephant fish is omitted because there is no evidence that this region is present in the current assembly. The spotted gar is used as the osteichthyes representative, as it retained both copies of *GbX* and had no further whole genome duplications.

Supplementary Figure 4

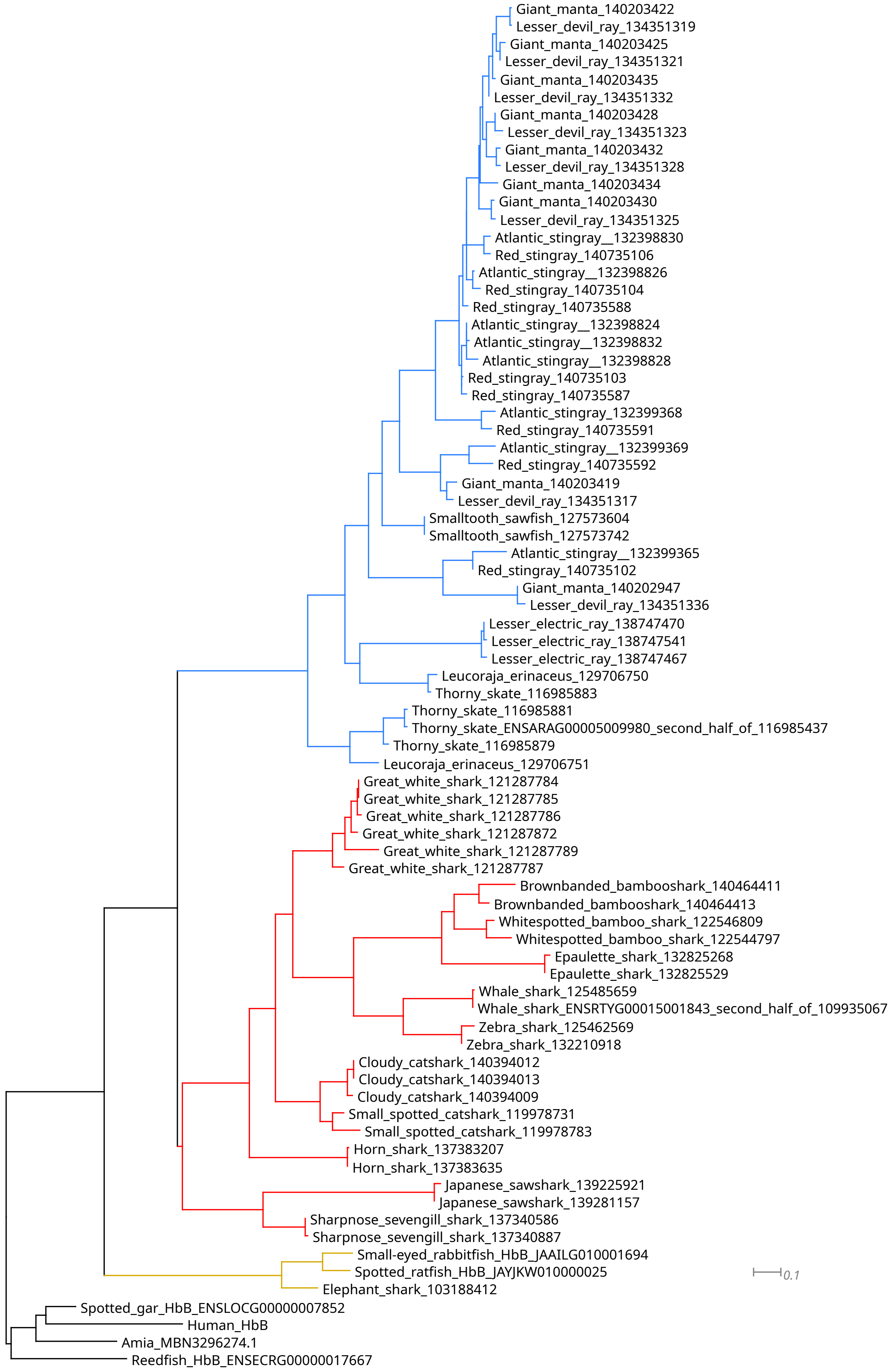

Supplementary Figure 4. Maximum likelihood phylogram depicting relationships among the β-globin genes of cartilaginous fishes forcing forcing each of Holocephali, Batoidea and Selachimorpha sequences to be monophyletic. Shark branches in red, batoid branches in blue and holostei branches in fucsia. The tree was rooted using bony vertebrate β-globins.

Supplementary Figure 5

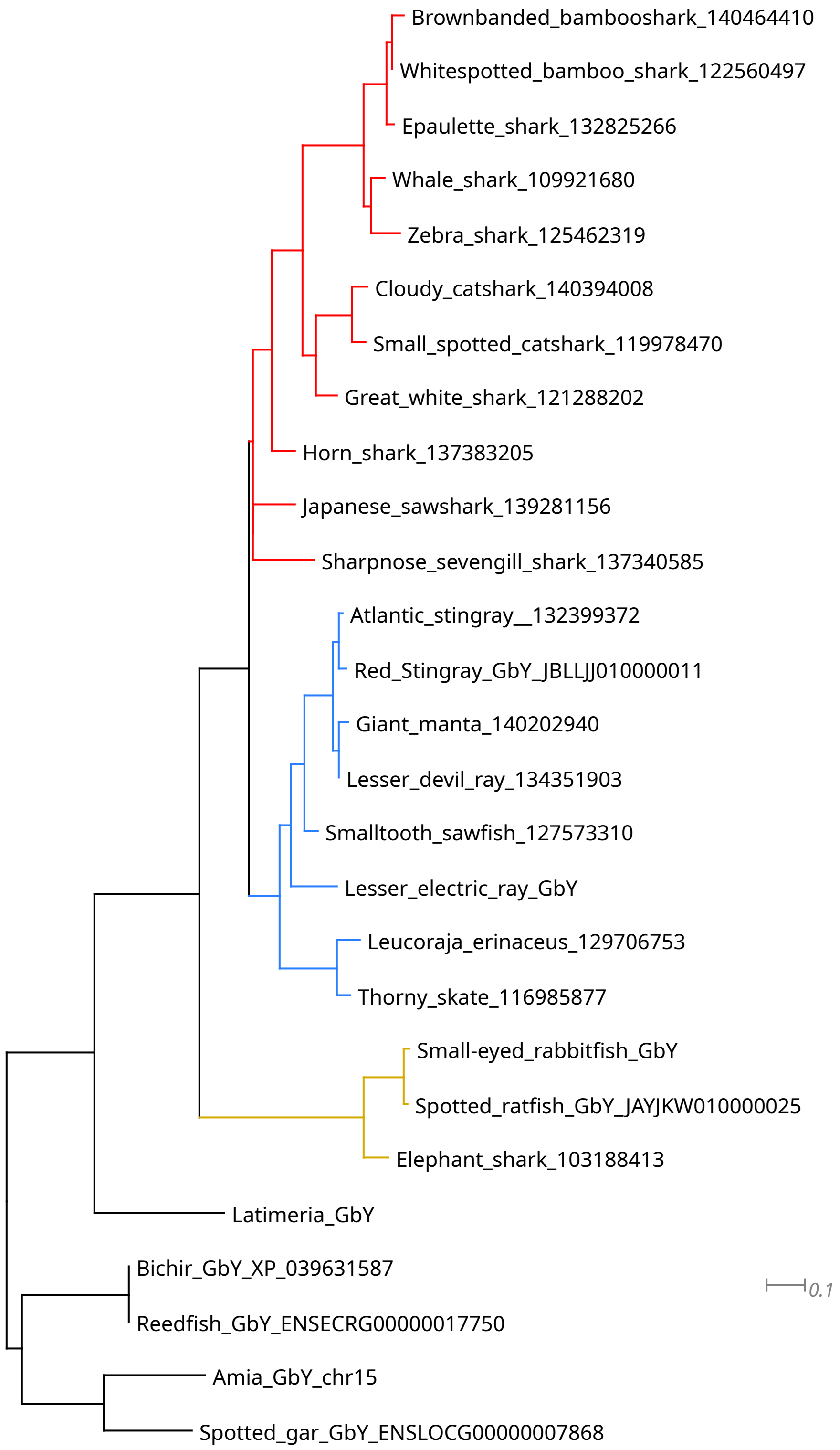

Supplementary Figure 5. Maximum likelihood phylogram depicting relationships among the GbY genes of cartilaginous fishes forcing forcing each of Holocephali, Batoidea and Selachimorpha sequences to be monophyletic. The tree was rooted using bony vertebrate GbY protein sequences. Shark branches in red, batoid branches in blue and holostei branches in fuchsia.

Supplementary Figure 6

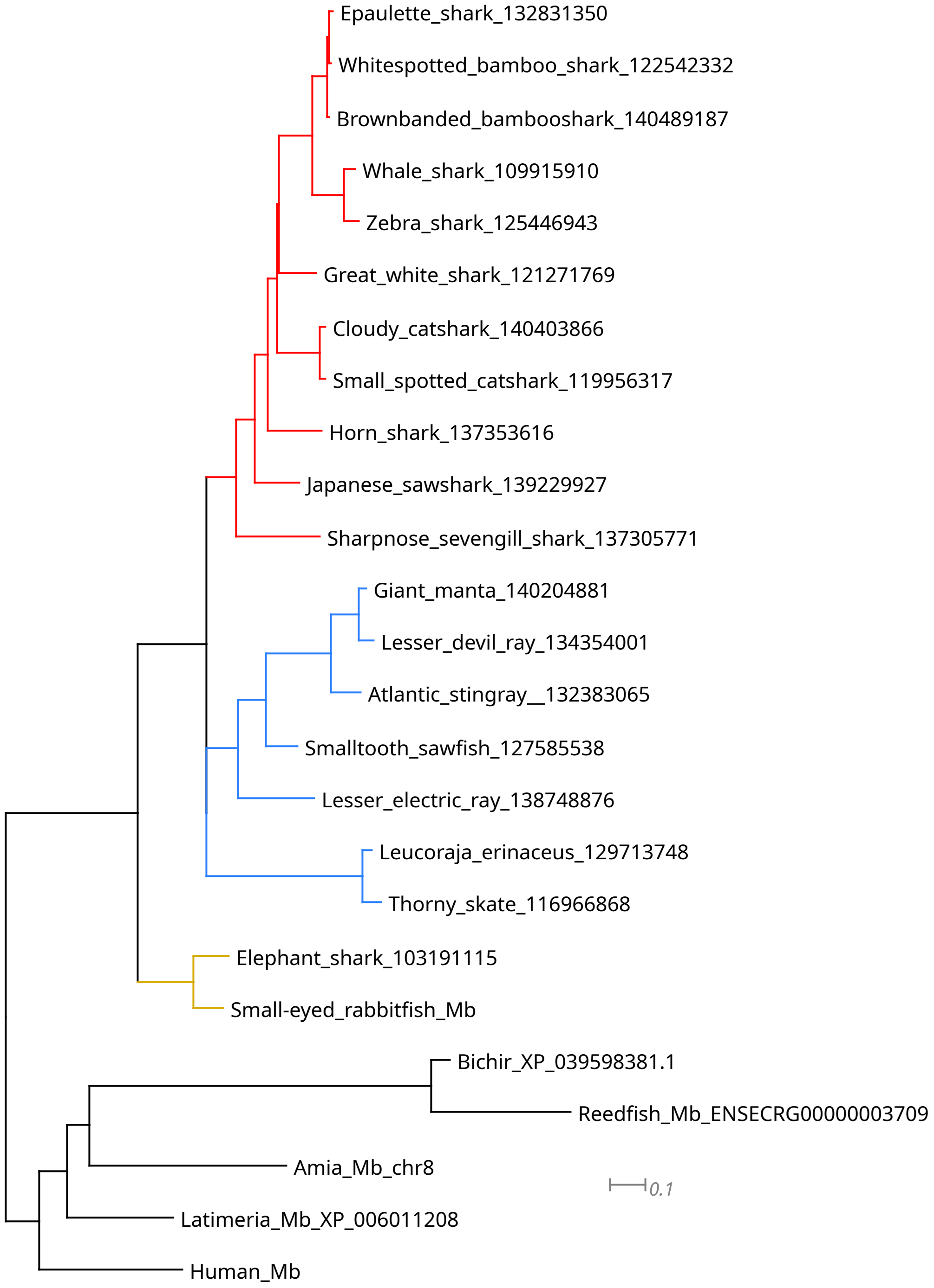

Supplementary Figure 6. Maximum likelihood phylogram depicting relationships among the Mb protein sequences of cartilaginous fishes forcing forcing each of Holocephali, Batoidea and Selachimorpha sequences to be monophyletic. The tree was rooted using the Mb proteins of bony vertebrates. Shark branches in red, batoid branches in blue and holostei branches in fuchsia.

Supplementary Figure 7

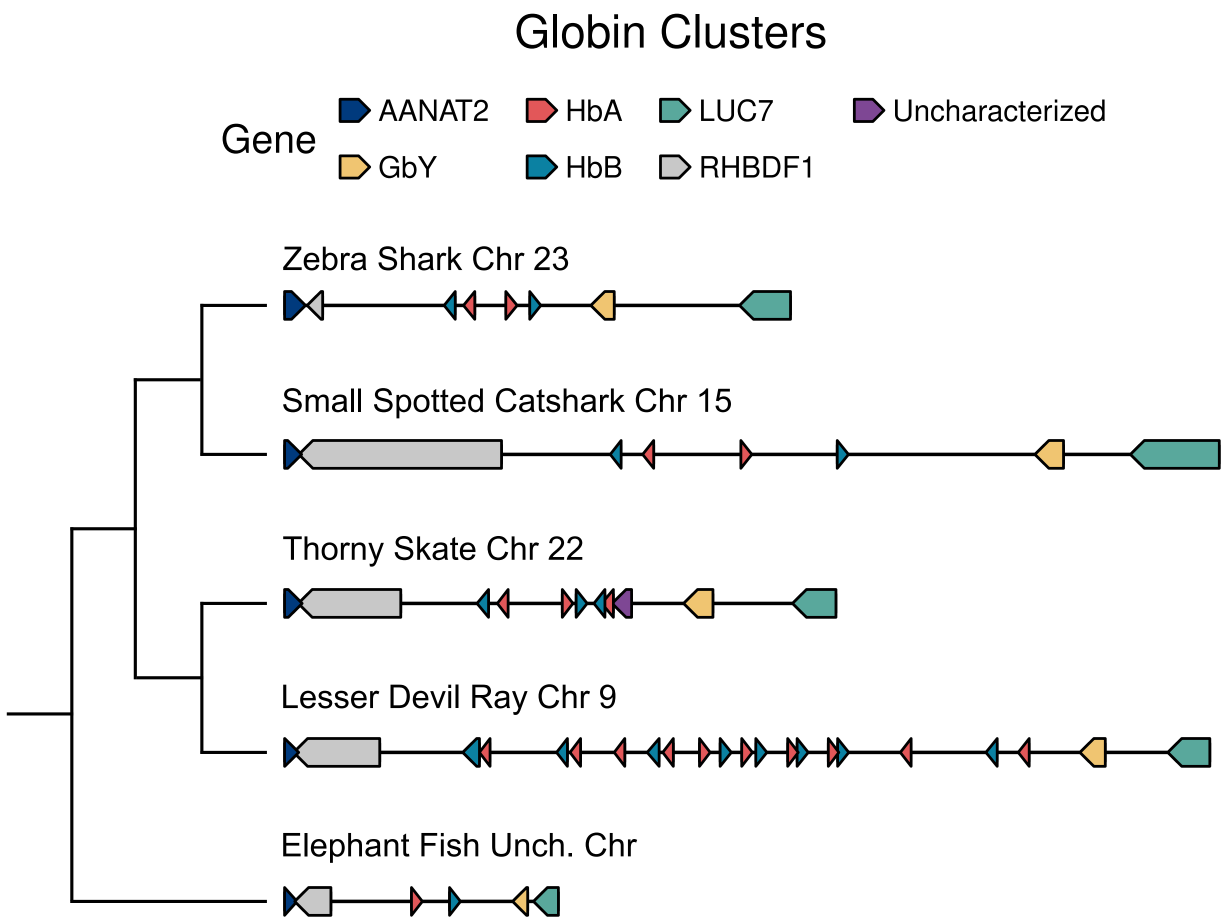

Supplementary Figure 7. Synteny of hemoglobin clusters and GbY. Two representative taxa from the Selachimorpha and the Batoidea were chosen for visual clarity. Only one representative from the Holocephali is shown as it is the only species with a RefSeq genome

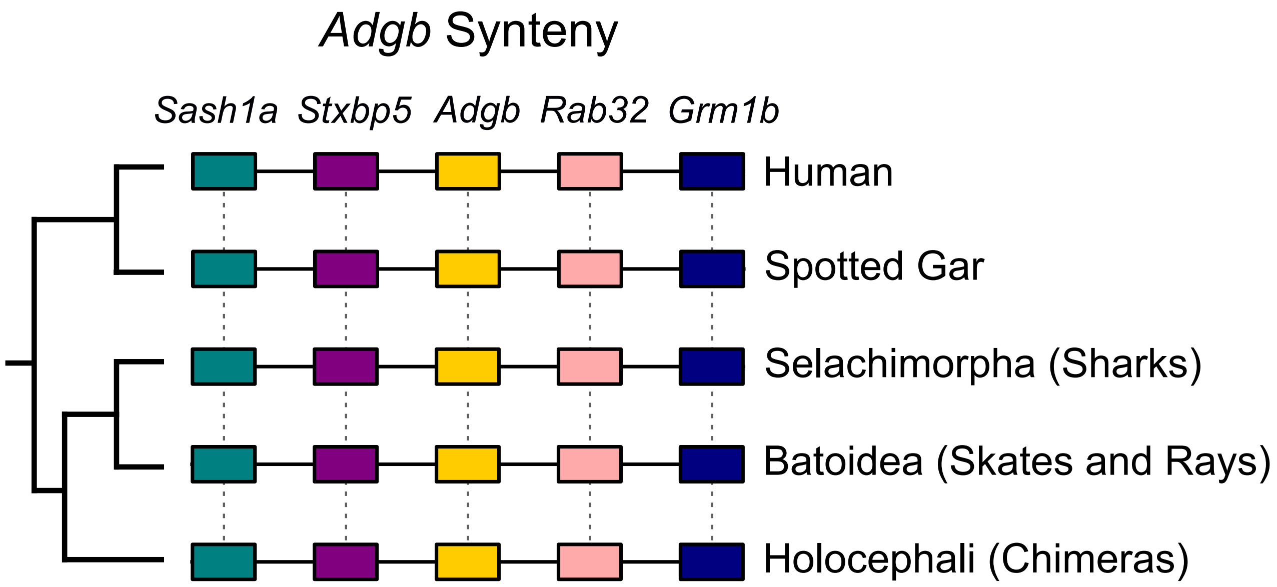

Supplementary Figure 8. The synteny of *Adgb* is conserved across cartilaginous and bony vertebrates. Schematic of generalized *Adgb* synteny with phylogentic realationships are shown by the dendrogram to the left.

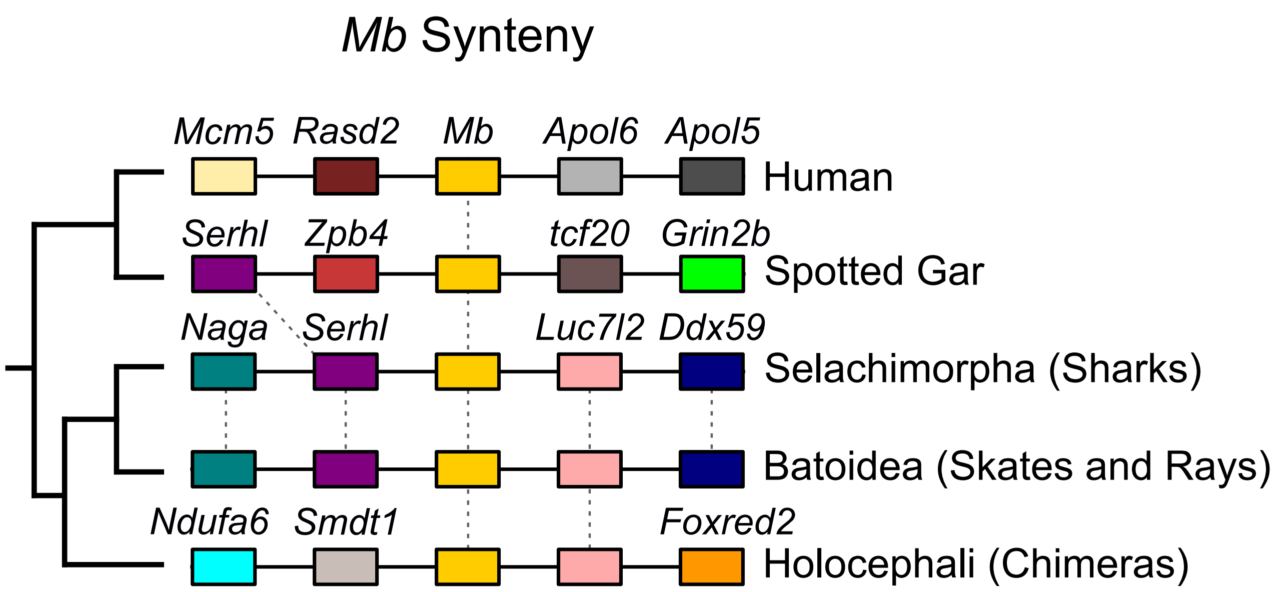

Supplementary Figure 9. The synteny of *Mb* is conserved across cartilaginous fish and the spotted gar, but not with humans. Schematic of generalized *Mb* synteny with phylogentic realationships are shown by the dendrogram to the left.

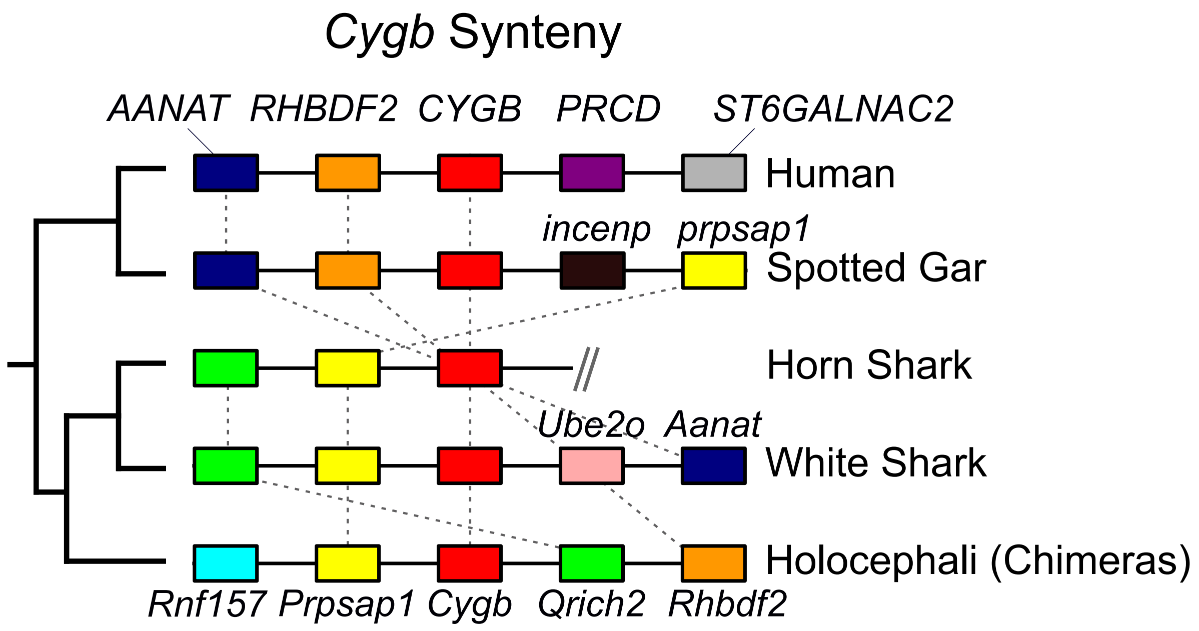

Supplementary Figure 10. The synteny of *Cygb* is conserved across cartilaginous fish and bony vertebrates. Orthologous genes are found in close proximity to *Cygb*, suggesting that cartilagionous fish *Cygb* is in its ancestral location. Phylogentic relationships are shown by the dendrogram to the left. The horn shark contig with *Cygb* is fractionated, indicated by the gray parallel lines.

Supplementary figure 11

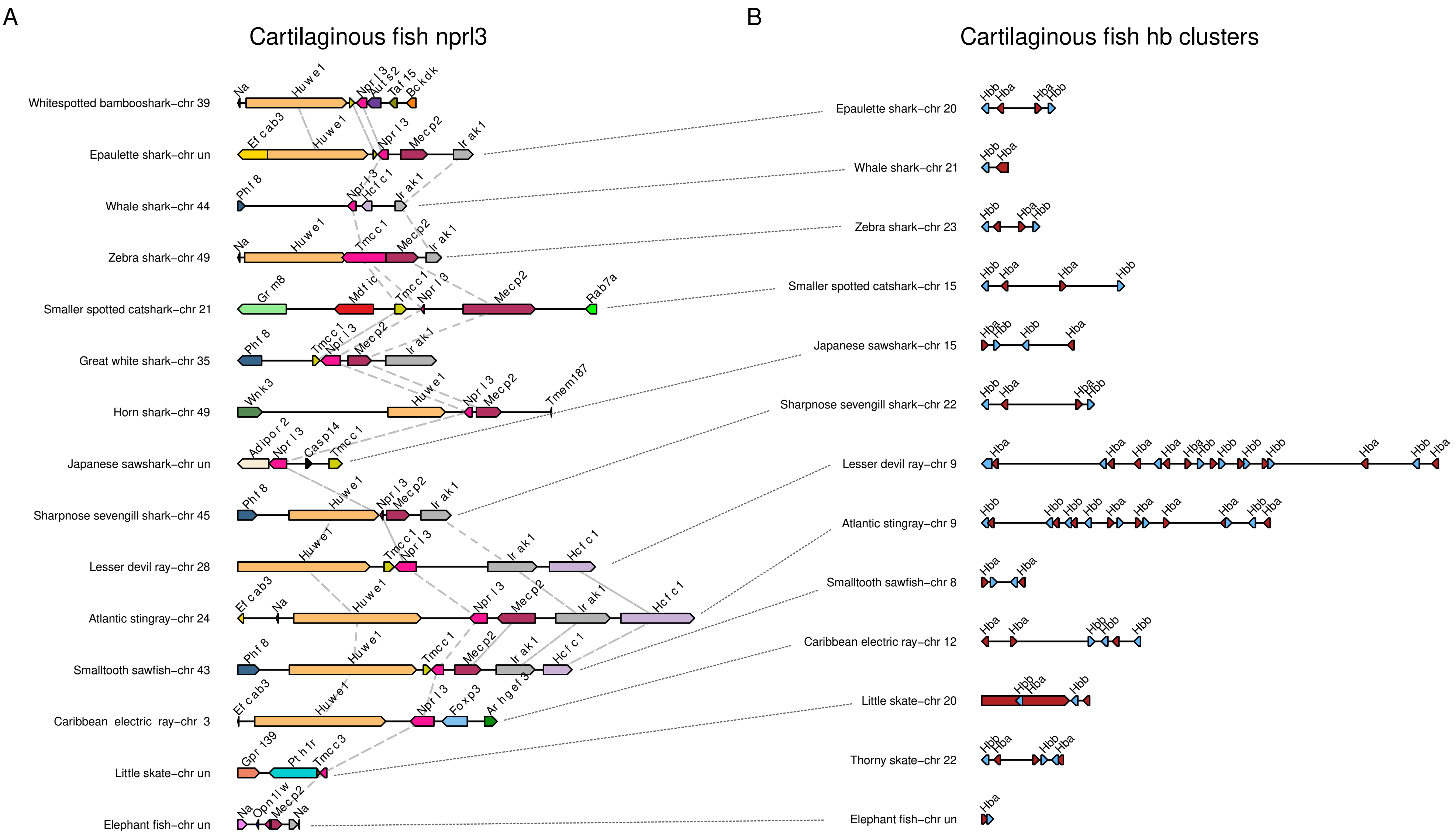

Supplementary figure 11. Cartilaginous fish *Nprl3* and Hb clusters are on different chromosomes. Species are organized by the phylogenetic arrangment presented in fig. 1. Dashed lines connect the same taxa that appear in panels a and b. Taxa were left out when their genome assemblies were too fragmented to be informative, or if the scale made their schematic difficult to interpret.

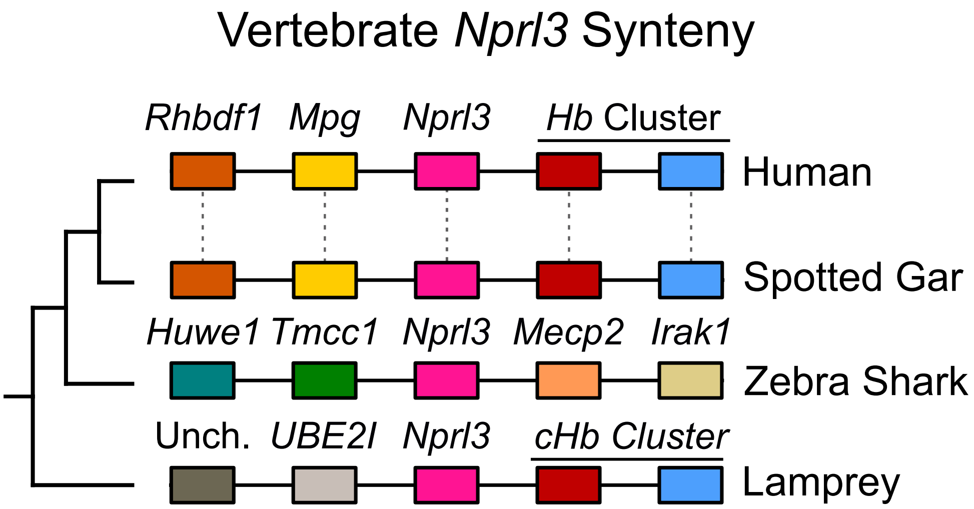

Supplementary Figure 12. *Nprl3* of cartilaginous fish is not syntenic with other major vertebrate clades. In jawless fish and bony fish, *Nprl3* flanks hemoglobin clusters and its genic region encodes major regulatory elements of hemoglobin expression. The hemoglobin clusters of cartilaginous fish appear on different crhomosomes than *Nprl3*.

Supplementary Figure 13

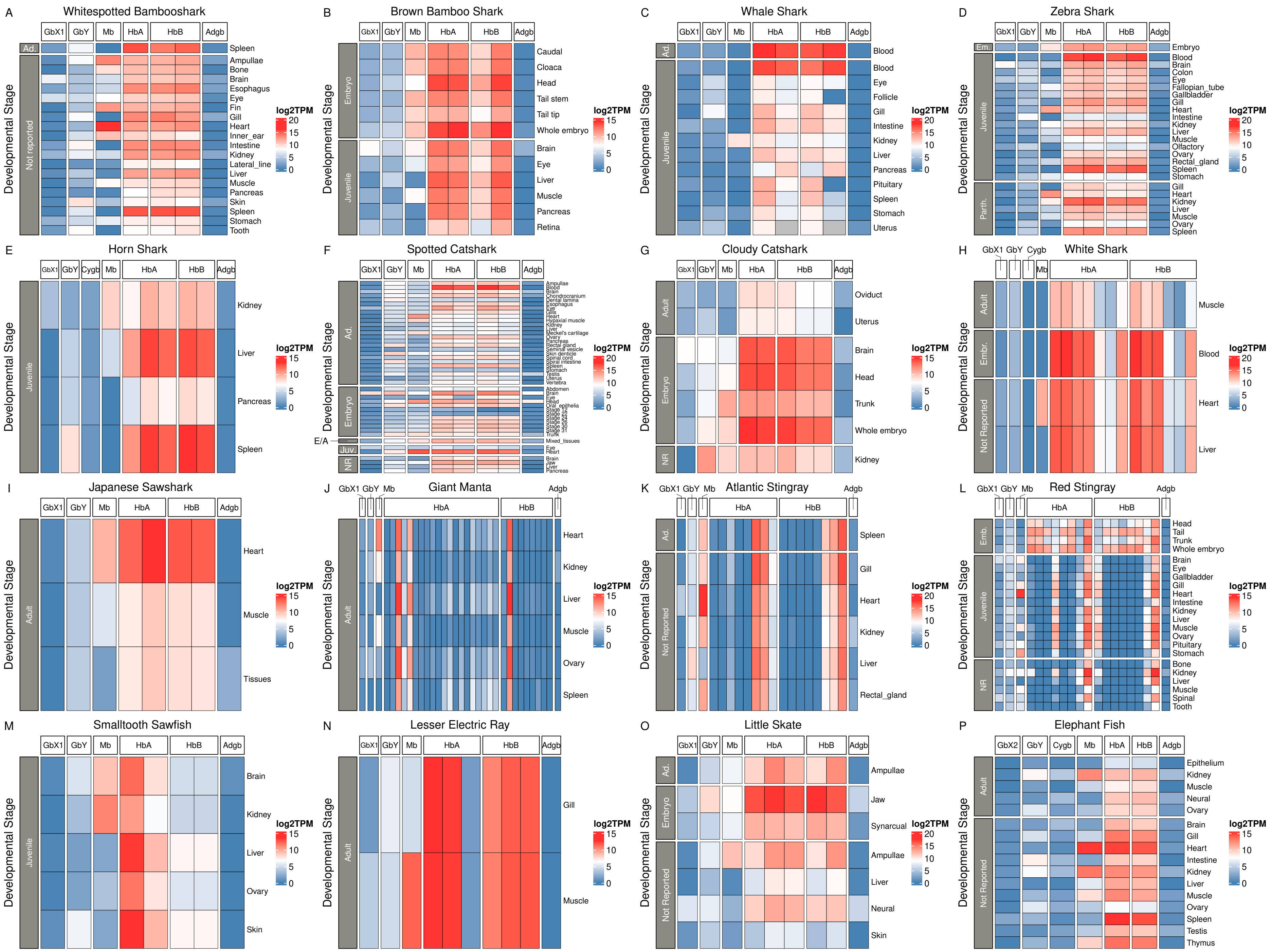

Supplementary Figure 13. Globin expression of cartilaginous fish across tissues and developmental stages. Expression estimates were derived from log2 transformed TPM values assigned by Kallisto. Tissues and developmental stages were labeled according to information in the SRA run selector metadata. Em. = Embryo, Ad. = Adult, NR = Not Reported, E/A = Embryo/Adult.
